# Supplementary material for: Structural basis for activation and gating of IP3 receptors
Source: Nat Commun. 2022 Mar 17;13:1408. doi: 10.1038/s41467-022-29073-2 (PMC8930994; doi:10.1038/s41467-022-29073-2)
Supplement: Supplementary file 1 — Supplementary Information [file 41467_2022_29073_MOESM1_ESM.pdf]

Supplementary Information:

Structural basis for activation and gating of IP_3 receptors

Emily A. Schmitz, Hirohide Takahashi, Erkan Karakas

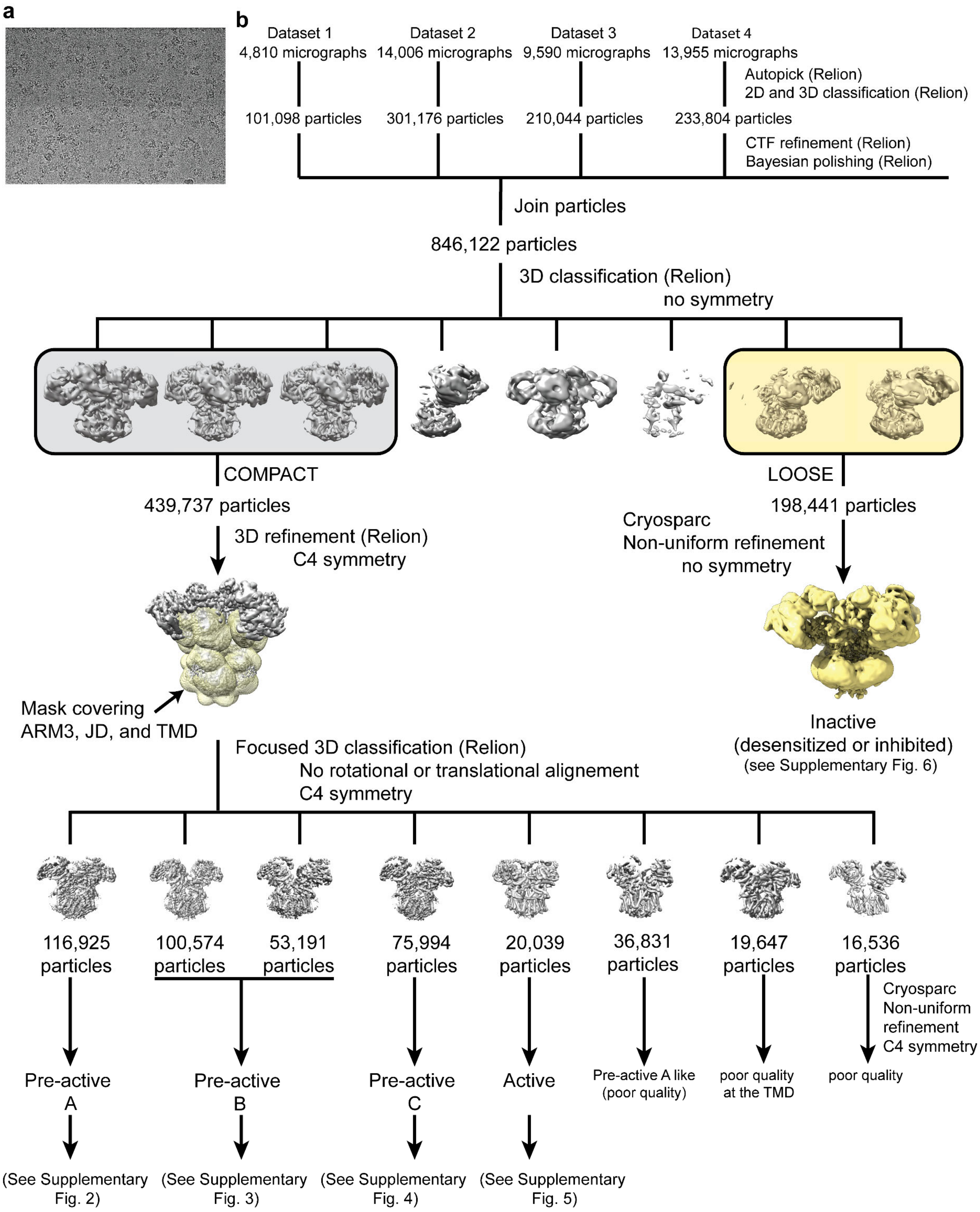

Supplementary Fig. 1: Cryo-EM analysis of hIP3R-3. **a** Representative cryo-EM micrograph of the hIP_3R-3 dataset. **b** Flowchart detailing the particle selection and refinement procedure to obtain the cryo-EM maps of hIP_3R-3 in different gating conformations. The structure in the inactive conformation (yellow) is low-pass filtered using a local resolution map. See methods for details.

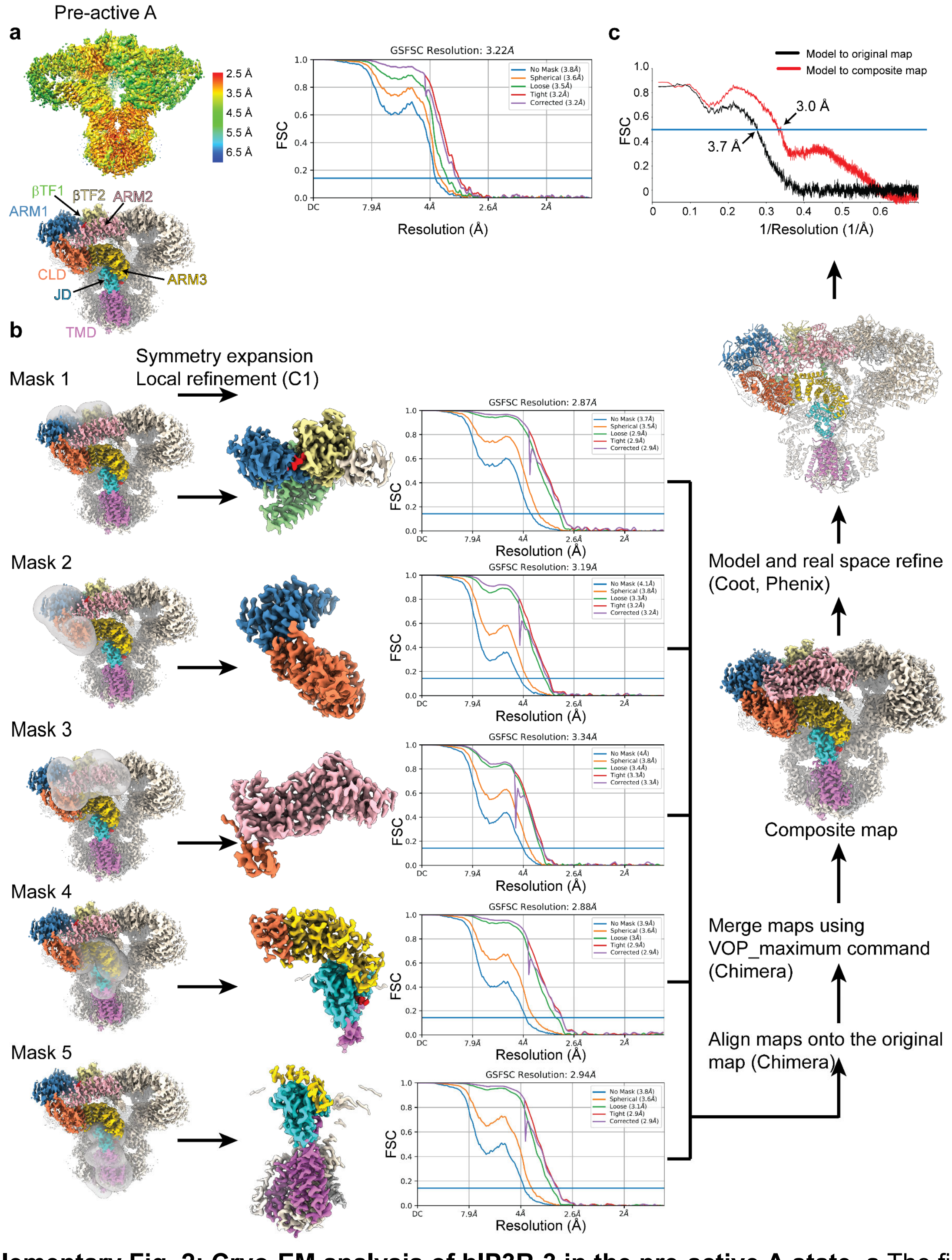

Supplementary Fig. 2: Cryo-EM analysis of hIP3R-3 in the pre-active A state. a The final refined 3D reconstructions of hIP3R-3 colored by local resolution (top) and individual domains (bottom) as in Fig. 1. FSC curve after Non-uniform refinement in CryoSPARC is shown. **b** Diagram showing the local refinement strategy. Masks are shown transparent, and domains are colored as in Fig.1. Arrows point to the maps obtained after local refinement. FSC curves after local refinement in CryoSPARC are shown for each mask. See methods for details. **c** FSC curves of the refined model versus the original EM map (black) and the composite map (red) are shown.

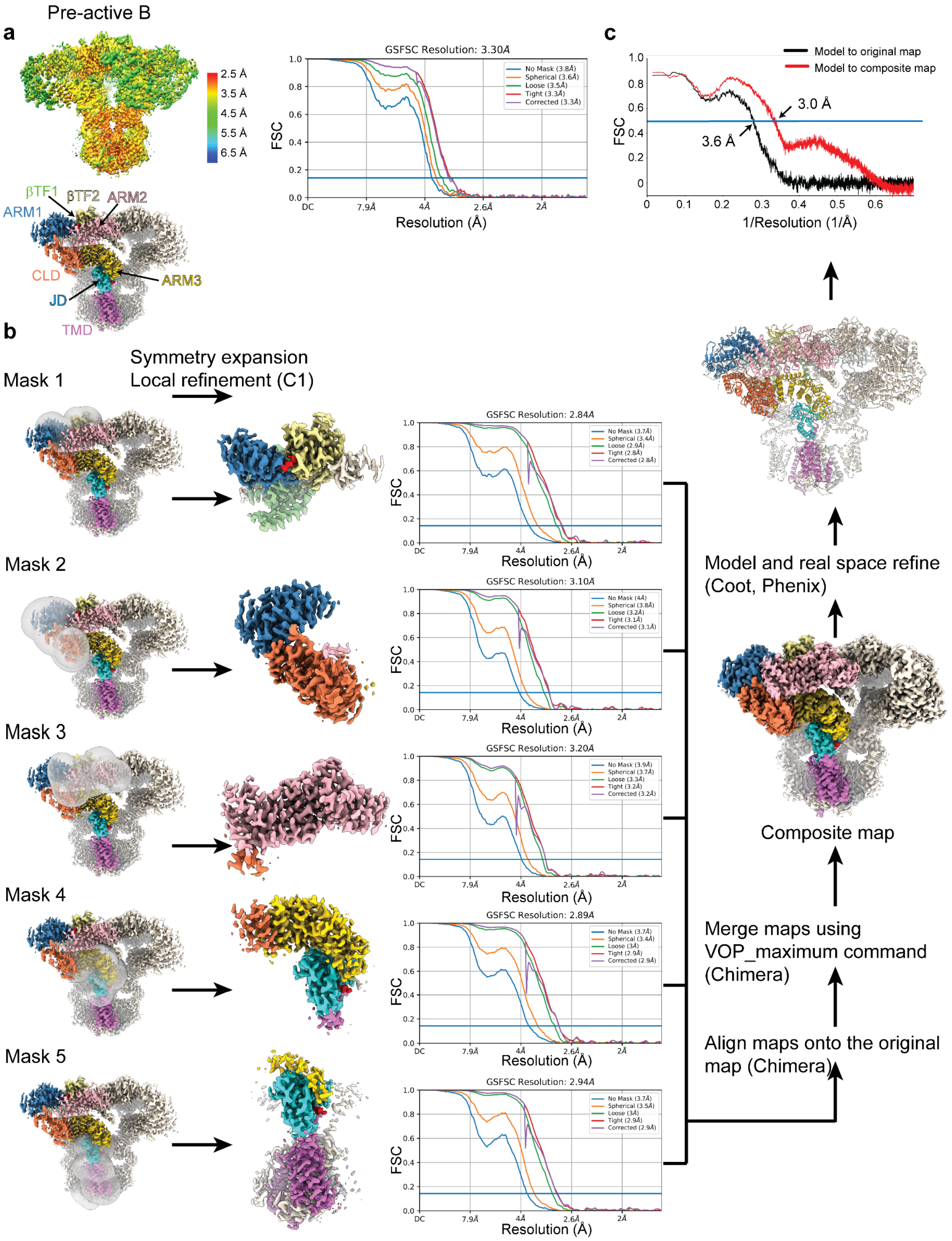

Supplementary Fig. 3: Cryo-EM analysis of hIP3R-3 in the pre-active B state. a The final refined 3D reconstructions of hIP3R-3 colored by local resolution (top) and individual domains (bottom) as in Fig. 1. FSC curve after Non-uniform refinement in CryoSPARC is shown. **b** Diagram showing the local refinement strategy. Masks are shown transparent, and domains are colored as in Fig.1. Arrows point to the maps obtained after local refinement. FSC curves after local refinement in CryoSPARC are shown for each mask. See methods for details. **c** FSC curves of the refined model versus the original EM map (black) and the composite map (red) are shown.

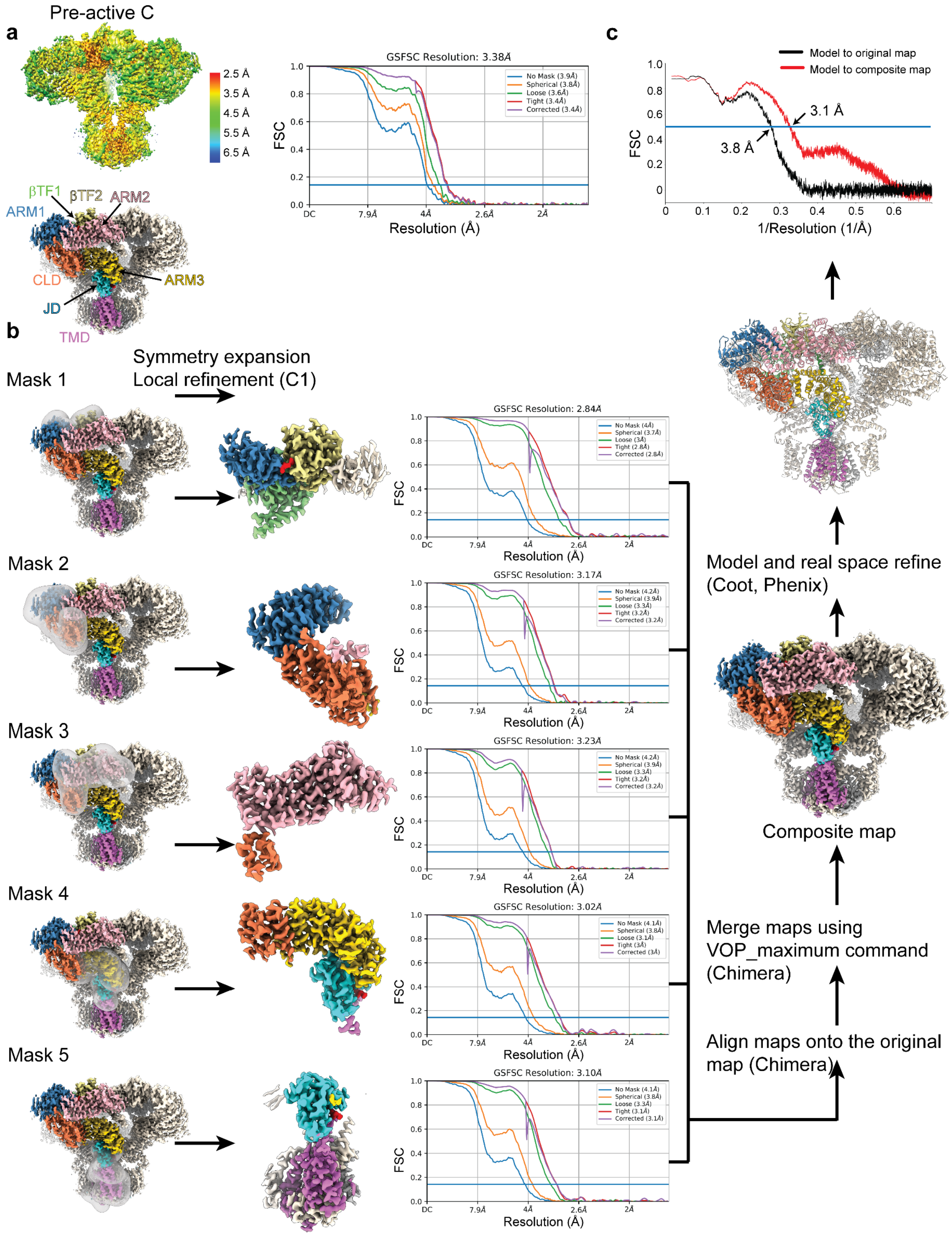

Supplementary Fig. 4: Cryo-EM analysis of hIP3R-3 in the pre-active C state. a The final refined 3D reconstructions of hIP3R-3 colored by local resolution (top) and individual domains (bottom) as in Fig. 1. FSC curve after Non-uniform refinement in CryoSPARC is shown. **b** Diagram showing the local refinement strategy. Masks are shown transparent, and domains are colored as in Fig.1. Arrows point to the maps obtained after local refinement. FSC curves after local refinement in CryoSPARC are shown for each mask. See methods for details. **c** FSC curves of the refined model versus the original EM map (black) and the composite map (red) are shown.

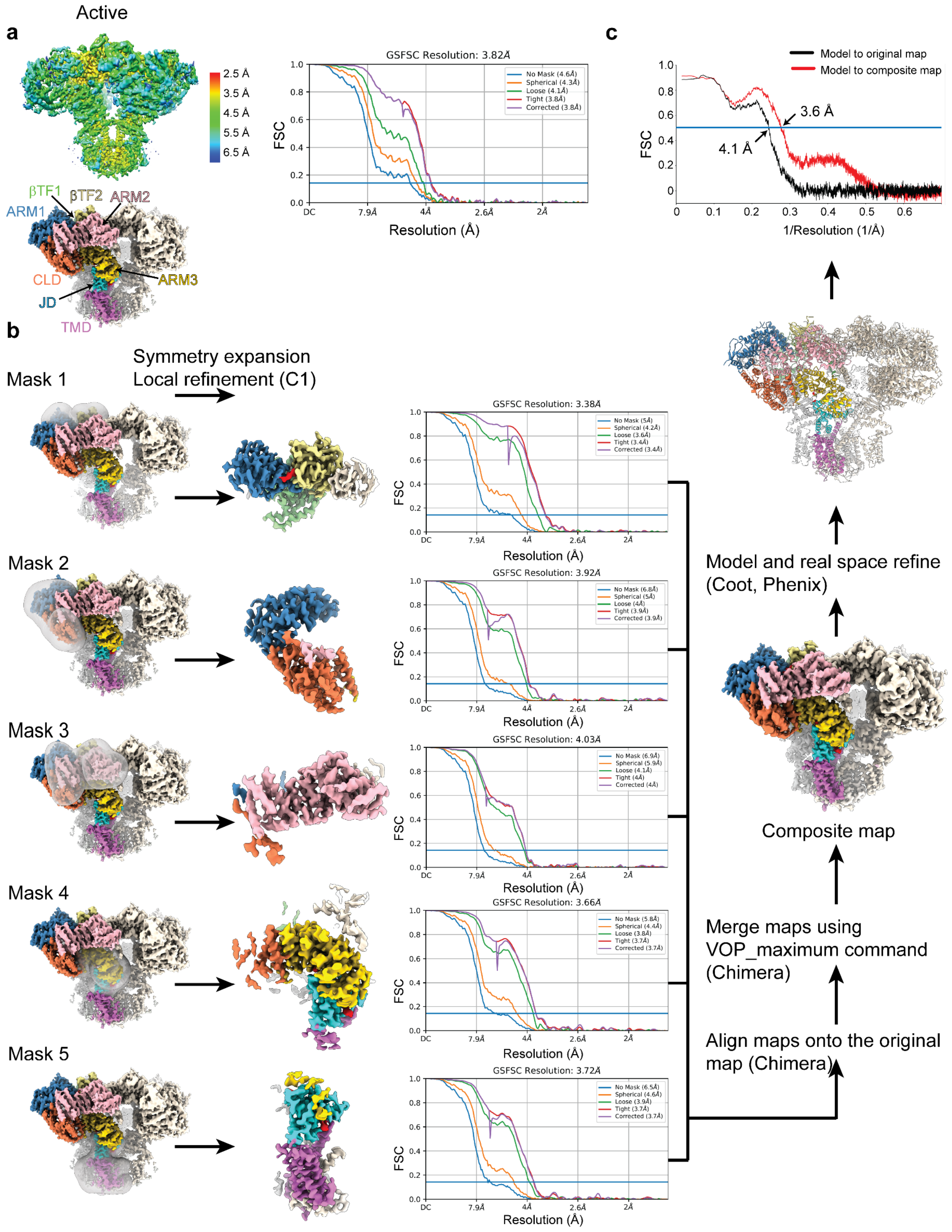

Supplementary Fig. 5: Cryo-EM analysis of hIP3R-3 in the active state. a The final refined 3D reconstructions of hIP3R-3 colored by local resolution (top) and individual domains (bottom) as in Fig. 1. FSC curve after Non-uniform refinement in CryoSPARC is shown. **b** Diagram showing the local refinement strategy. Masks are shown transparent, and domains are colored as in Fig.1. Arrows point to the maps obtained after local refinement. FSC curves after local refinement in CryoSPARC are shown for each mask. See methods for details. **c** FSC curves of the refined model versus the original EM map (black) and the composite map (red) are shown.

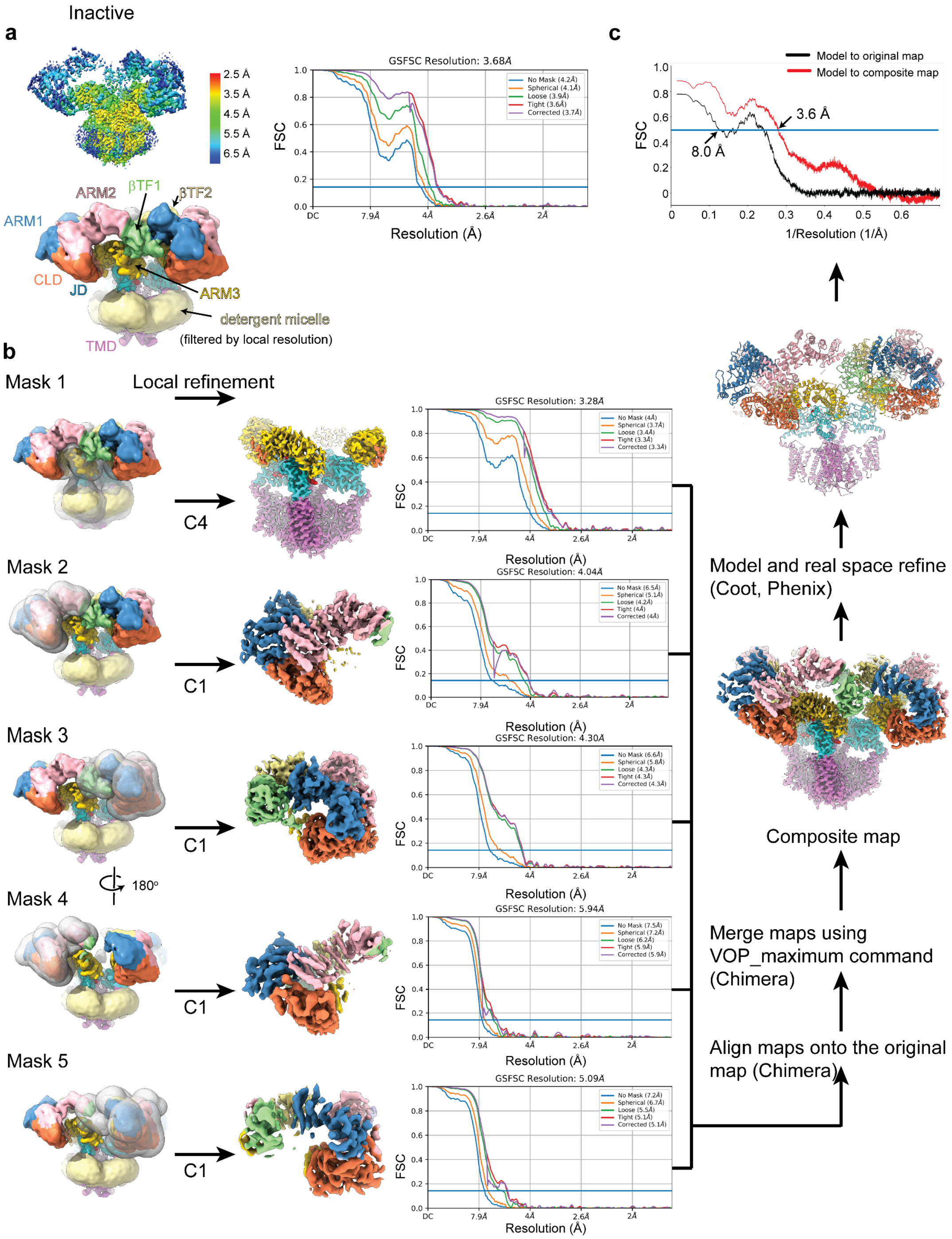

Supplementary Fig. 6: Cryo-EM analysis of hIP3R-3 in the inactive state. a The final refined 3D reconstructions of hIP3R-3 colored by local resolution (top) and individual domains (bottom) as in Fig. 1. The map colored by individual domains is low-pass filtered using the local resolution map. FSC curve after Non-uniform refinement in CryoSPARC is shown. **b** Diagram showing the local refinement strategy. Masks are shown transparent, and domains are colored as in Fig.1. Arrows point to the maps obtained after local refinement. FSC curves after local refinement in CryoSPARC are shown for each mask. See methods for details. **c** FSC curves of the refined model versus the original EM map (black) and the composite map (red) are shown.

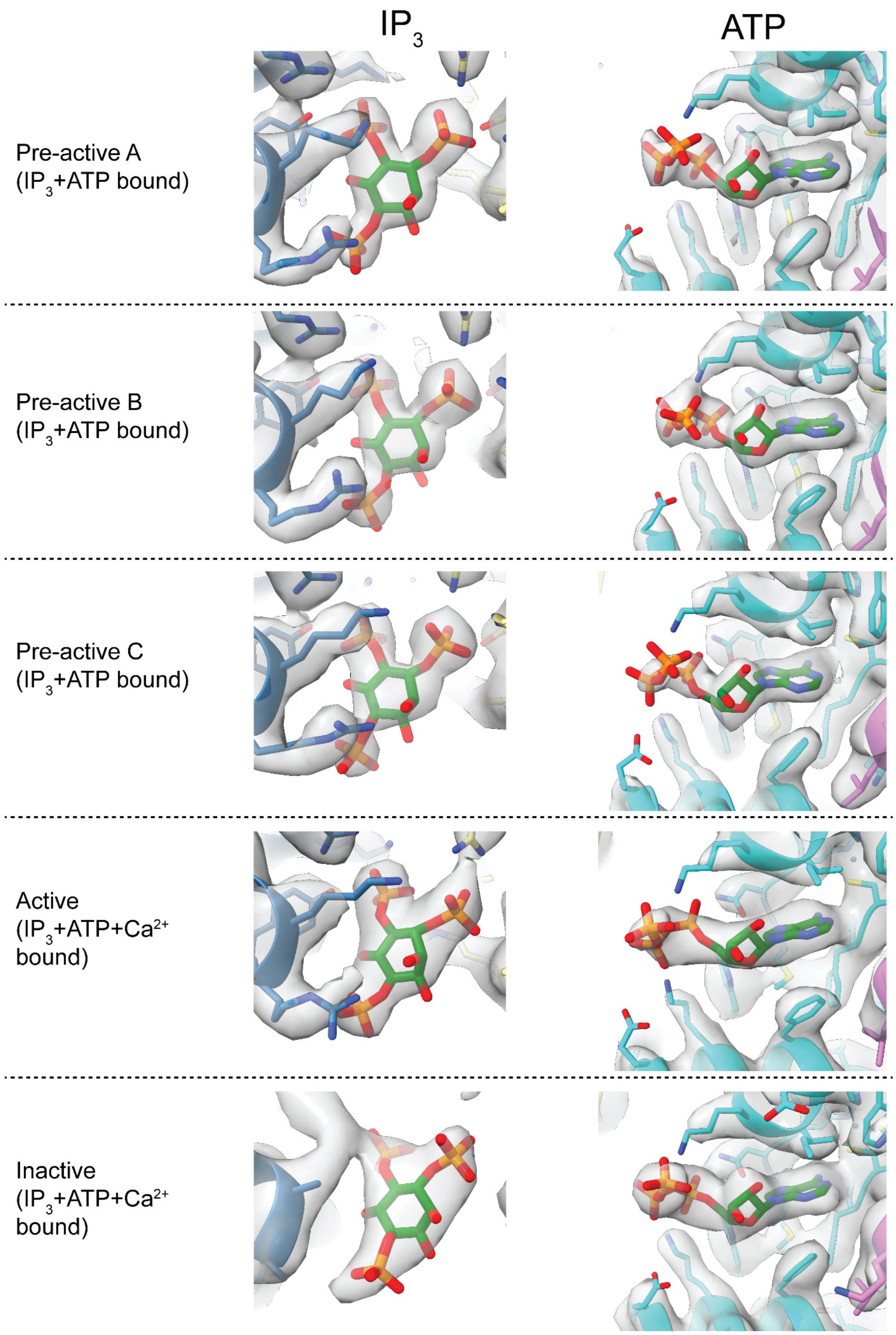

Supplementary Fig. 7: Ligand binding sites. Density maps for the IP_3 and ATP binding sites obtained through local refinements are shown as transparent grey surfaces. The ligands and the neighboring residues are represented as sticks.

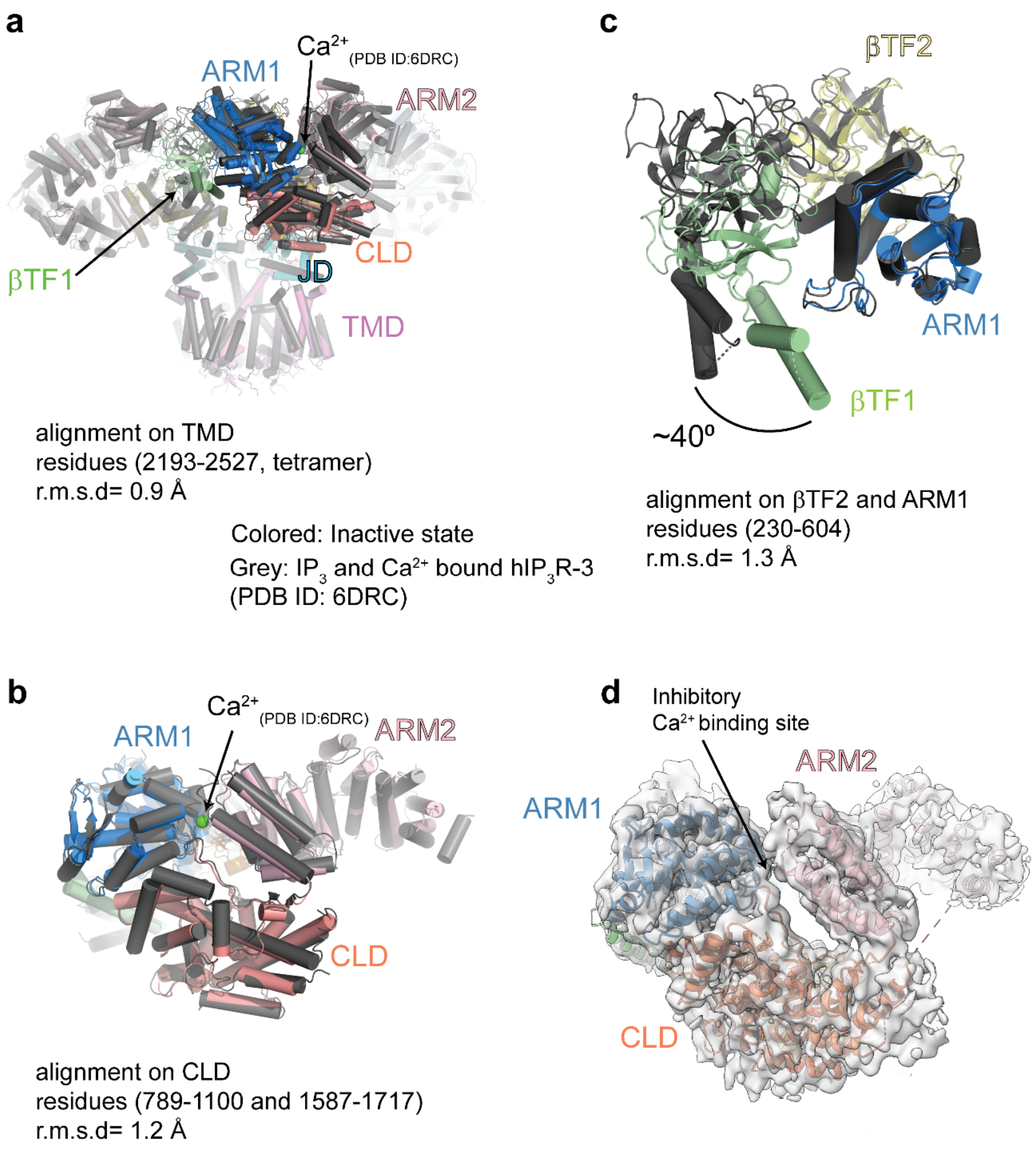

Supplementary Fig. 8: Structural comparison of the hIP$_3$R-3 structures in the inactive and Ca^{2+} inhibited states. a-c Ribbon representations of hIP$_3$R-3 in the inactive state and the previously published IP$_3$ and Ca^{2+} bound hIP$_3$R-3 structure (colored as in Fig. 1) obtained in the presence of inhibitory Ca^{2+} concentrations (grey, PDB ID: 6DRC [1]) focusing on similarities in the overall structures (**a**) and the inhibitory Ca^{2+} binding site (**b**), and differences in the IP$_3$ binding core and βTF1 orientation (**c**). The aligned regions and the root mean square deviation (r.m.s.d) values are shown for each panel. The green sphere represents the Ca^{2+} modeled in 6DRC. **d** Cryo-EM map after local refinement (transparent gray) is shown around the ribbon representation of the ARM1, CLD, and ARM2 of the inactive structure. Although the quality of the map allows modeling the domains, the density for the side chains of the residues around the Ca^{2+} binding site is poorly resolved, hampering inspection of the presence of Ca^{2+}.

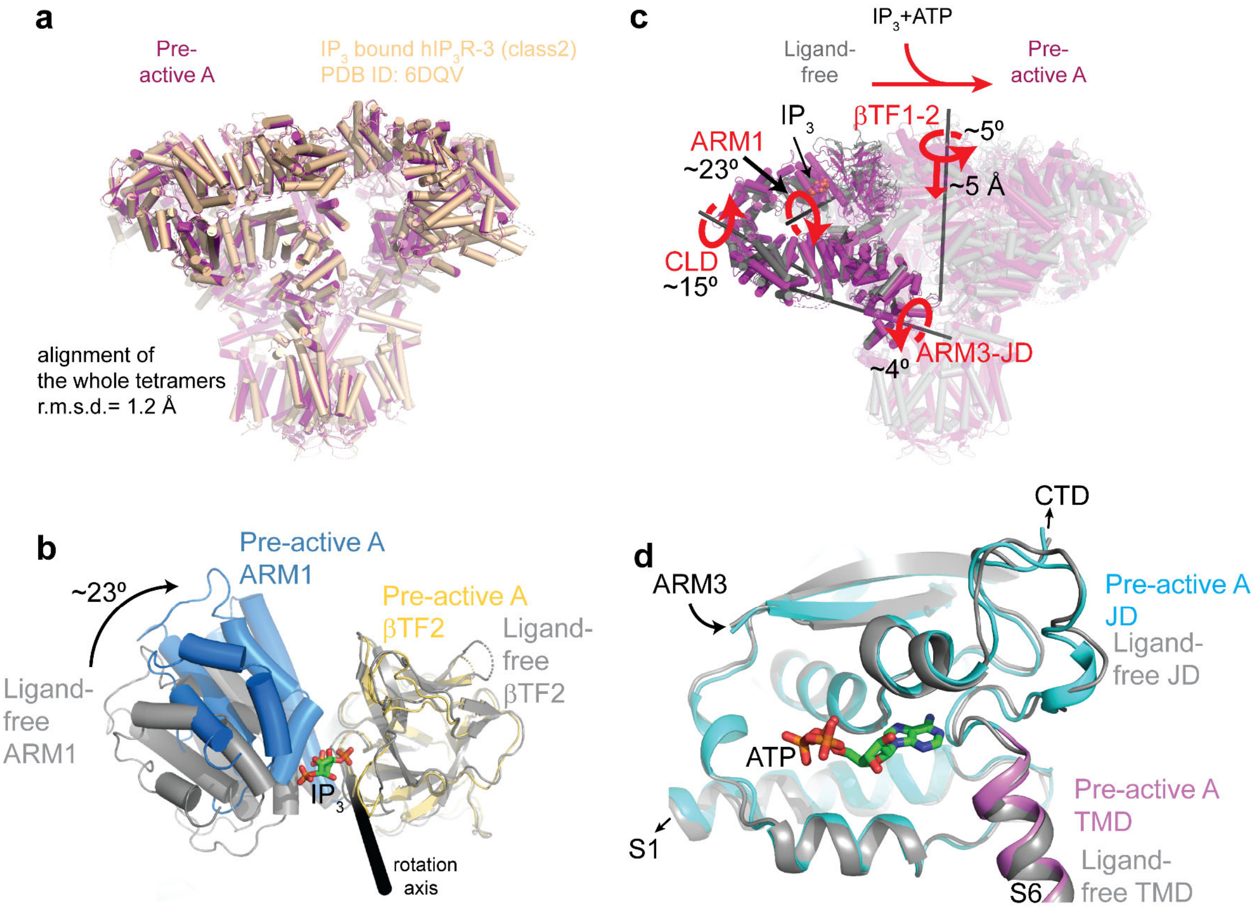

Supplementary Fig. 9: Structural comparison of the hIP$_3$R-3 structures in the ligand-free and Pre-active A conformations. **a** Ribbon representations of hIP$_3$R-3 in the pre-active A state and the previously published IP$_3$ bound class 2 state (PDB ID: 6DQV [1]). The intact receptors are used for alignment. **b** IP$_3$ induced conformational changes resulting in 23° rotation of the ARM1 relative to βTF2. The structures are aligned on the βTF2. Pre-active A structure is colored as in Fig.1, and Ligand-free structure is colored in grey. IP$_3$ is shown as green sticks. **c** Ribbon representations of hIP$_3$R structures in the resting (PDB ID: 6UQK [2]) superposed on the residues forming the selectivity filter and P-helix of the TMDs, emphasizing the conformational changes upon IP$_3$ and ATP binding. Domains with substantial conformational changes are shown in full colors only on one subunit, while the rest of the protein is transparent. Curved and straight red arrows indicate the rotation and translation of the domains with red labels relative to the rotation axis (black bars), respectively. **d** Close-up view of the ATP binding site. Pre-active A structure is colored as in Fig.1, and Ligand-free structure is colored in grey. ATP is shown as green sticks. No major conformational changes around the binding site are observed.

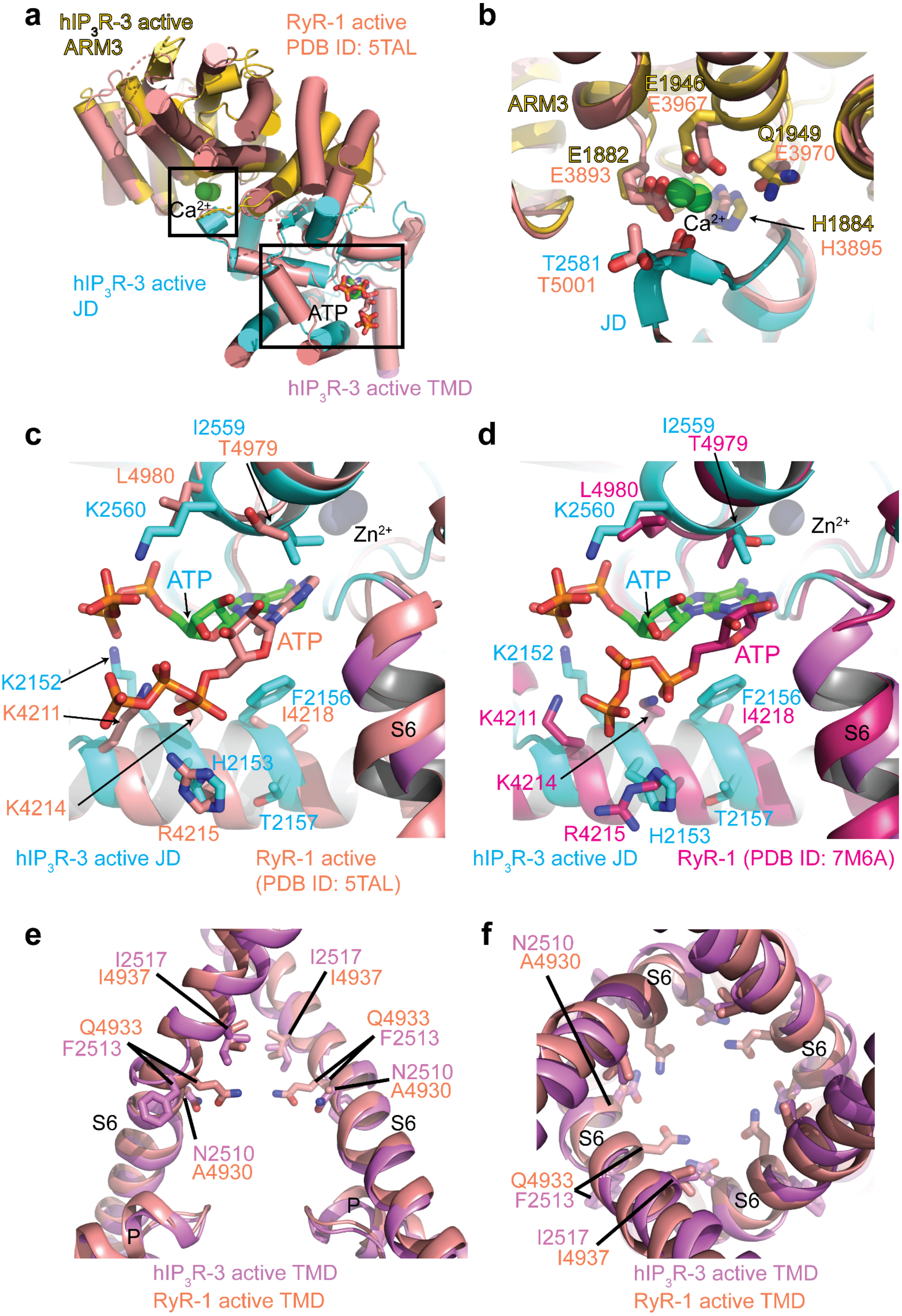

a
hIP3R-3 active ARM3
RyR-1 active PDB ID: 5TAL
Ca2+
hIP3R-3 active JD
ATP
hIP3R-3 active TMD
b
ARM3
E1946
E3967
Q1949
E3970
E1882
E3893
Ca2+
H1884
H3895
T2581
T5001
JD
c
I2559
T4979
L4980
K2560
Zn2+
ATP
ATP
K2152
K4211
F2156
I4218
S6
H2153
K4214
T2157
R4215
hIP3R-3 active JD
RyR-1 active (PDB ID: 5TAL)
d
I2559
T4979
L4980
K2560
Zn2+
ATP
K2152
ATP
F2156
K4211
I4218
S6
K4214
T2157
R4215
H2153
hIP3R-3 active JD
RyR-1 (PDB ID: 7M6A)
e
I2517
I4937
I2517
I4937
Q4933
F2513
Q4933
F2513
N2510
A4930
S6
S6
N2510
A4930
P
P
hIP3R-3 active TMD
RyR-1 active TMD
f
N2510
A4930
S6
S6
S6
Q4933
F2513
S6
I2517
I4937
hIP3R-3 active TMD
RyR-1 active TMD

Supplementary Fig. 10: Structural comparison of hIP$_3$R-3 and RyR1 structures in the active state. a An overlay of the ARM3, JD, and part of S6 of hIP$_3$R-3 with the corresponding domains of rabbit RyR-1 (PDB ID: 5TAL) [3]. hIP$_3$R-3 is colored as in Fig. 1, and RyR1 is colored in salmon. Ca^{2+} and ATP binding sites are boxed. **b** Close-up views of the Ca^{2+}. Residues coordinating Ca^{2+} are shown as sticks and labeled. **c-d** Close-up view of the ATP binding site of hIP$_3$R-3 aligned onto the corresponding domains of rabbit RyR-1s ((**c**) PDB ID: 5TAL and (**d**) PDB ID:7M6A [4]). Select residues are shown as sticks and labeled. **e-f** Alignment of the S6 and P helices viewed through the membrane plane (**e**) and cytoplasm (**f**). Two of the subunits were not shown in panel **e** for clarity.

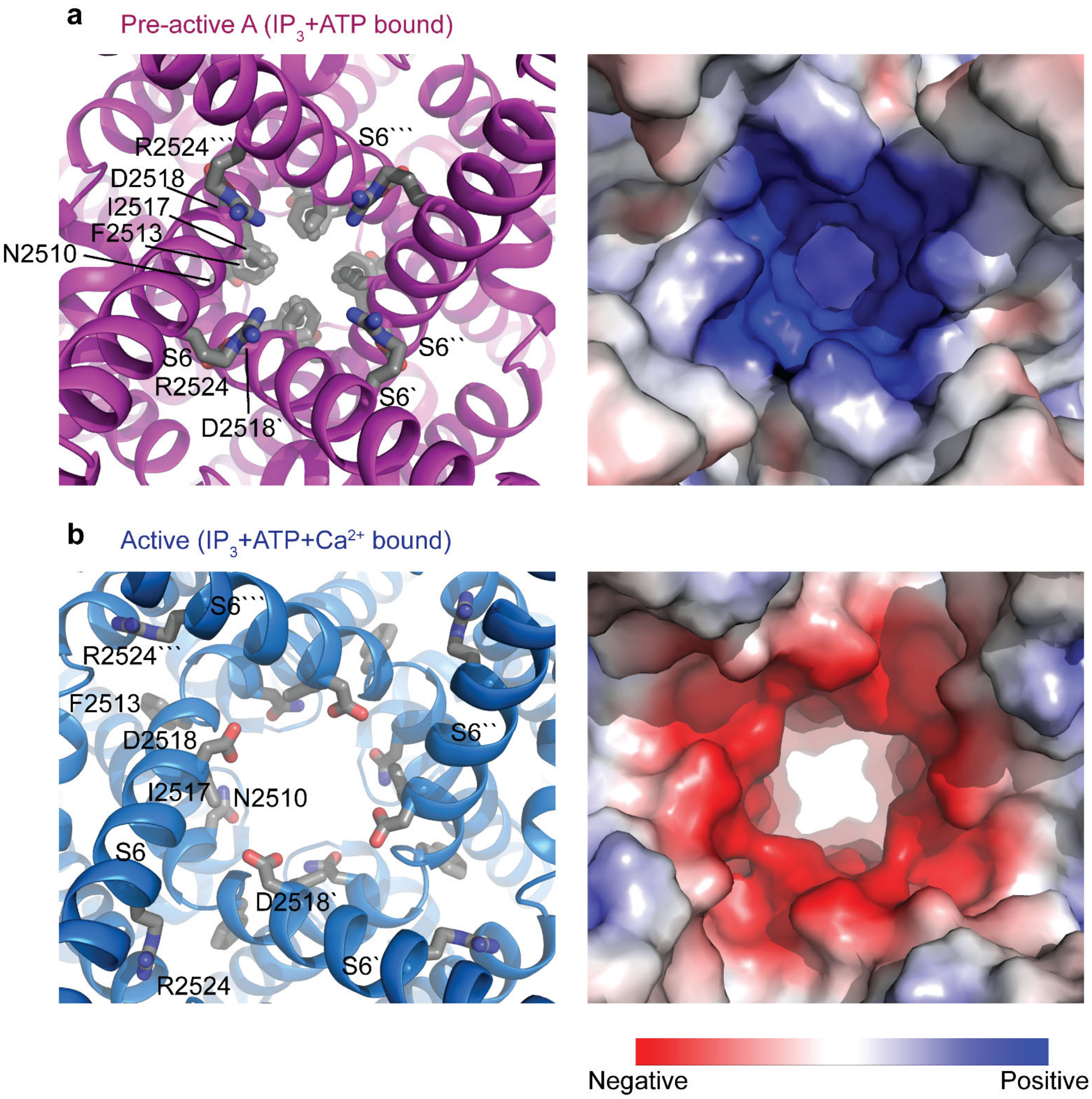

Supplementary Fig. 11: Structural comparison of hIP$_3$R-3 pores in closed and open conformations. a-b Close up cytoplasmic view of the IP$_3$R-3 pores in the closed (**a**) and open (**b**) conformations in ribbon (left) and electrostatic surface representations. Pore lining residues are shown as grey sticks. ` is used the differentiate residues in different subunits.

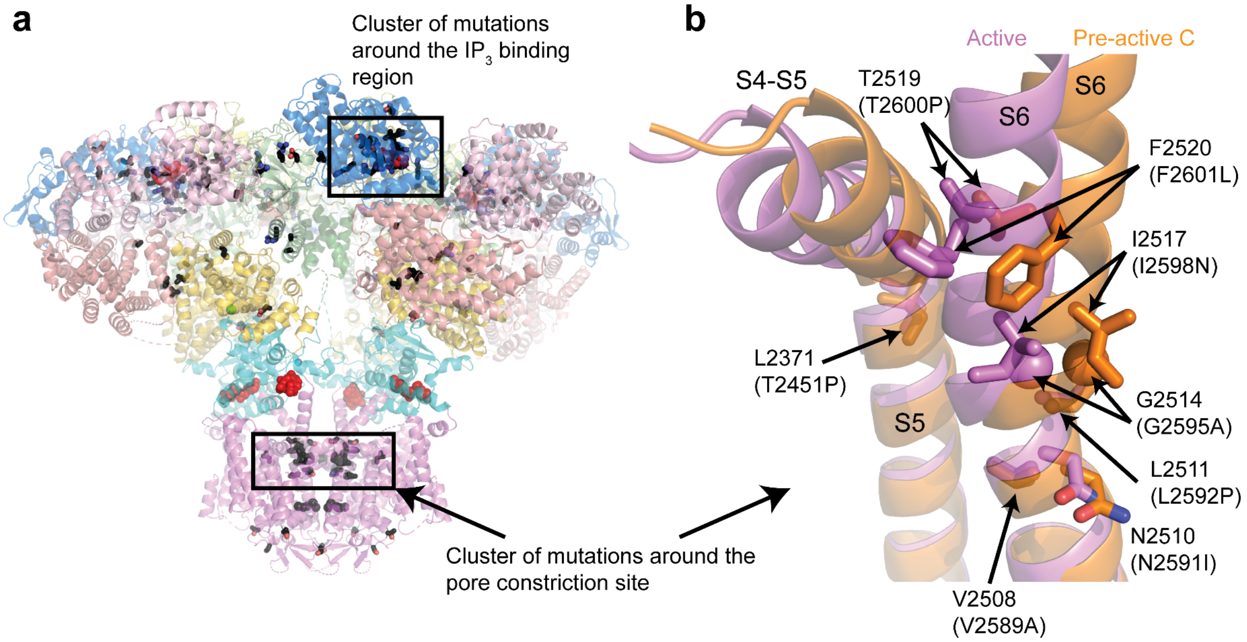

Supplementary Fig. 12: Mutation hot spots of IP_3Rs. a Mutations associated with diseases or annotated as pathogenic or likely pathogenic in ClinVar database (https://www.ncbi.nlm.nih.gov/clinvar/) for all three subtypes of IP_3Rs are mapped onto the hIP_3R-3 structure in the active conformation. Each domain is colored as in Fig. 1, and the mutated residues are shown as black sticks. IP_3 and ATP are shown as red spheres, and Ca^{2+} ions are shown as green spheres. Clusters of mutations around the IP_3 binding site and the pore constriction site are indicated in black boxes. **b** Close-up view of the mutated residues around the pore constriction site. Only one protomer of pre-active C and active state structures are shown. Structures are aligned as described in Fig. 2. Mutated residues are labeled based on IP_3R-3 numbering and IP_3R-1 numbering with the mutations inside parentheses. The reference sequences used for IP_3R-3 are UniProtKB-Q14573 and for IP_3R-1 are UniProtKB-Q14643.

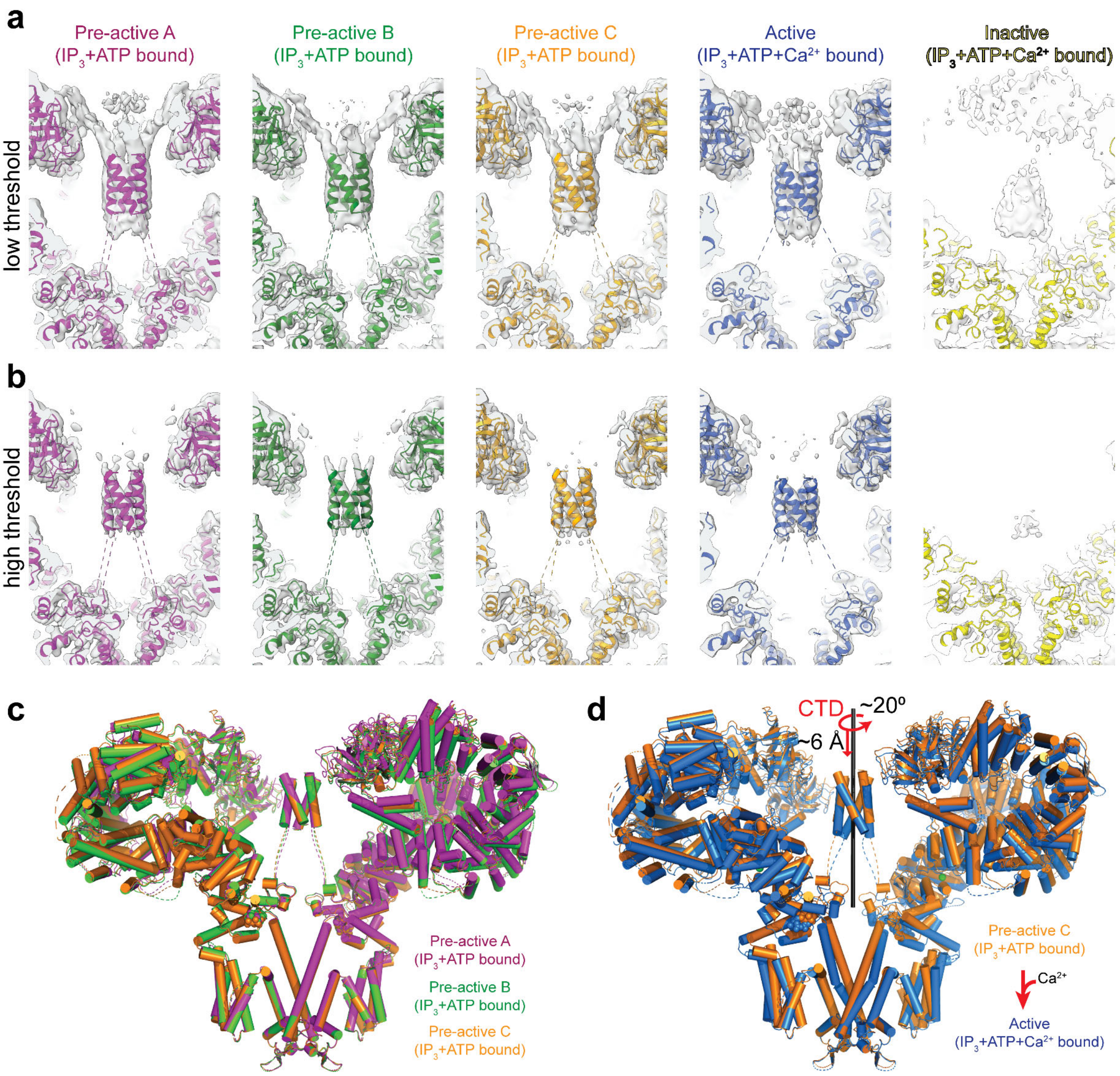

Supplementary Fig. 13: The flexible architecture of the CTD. a-b Unsharpened Cryo-EM map sections of hIP$_3$R-3 in different gating conformations are shown using lower (**a**) and higher (**b**) threshold values to emphasize the variability of the regions connecting the coiled-coil region to the B-TF1 and JDs. Maps are the original maps from the non-uniform refinements and are shown as transparent grey surfaces. The ribbon representations of the fitted models are colored as indicated above each column. **c** There are no noticeable changes for the coiled-coil region of hIP$_3$R-3 in the pre-active conformations. **d** The coiled coil region makes a screw-like motion towards the JD during activation. **c-d** Alignments are performed as in Figure 2. Only two opposing subunits are shown except for the coiled-coil region.

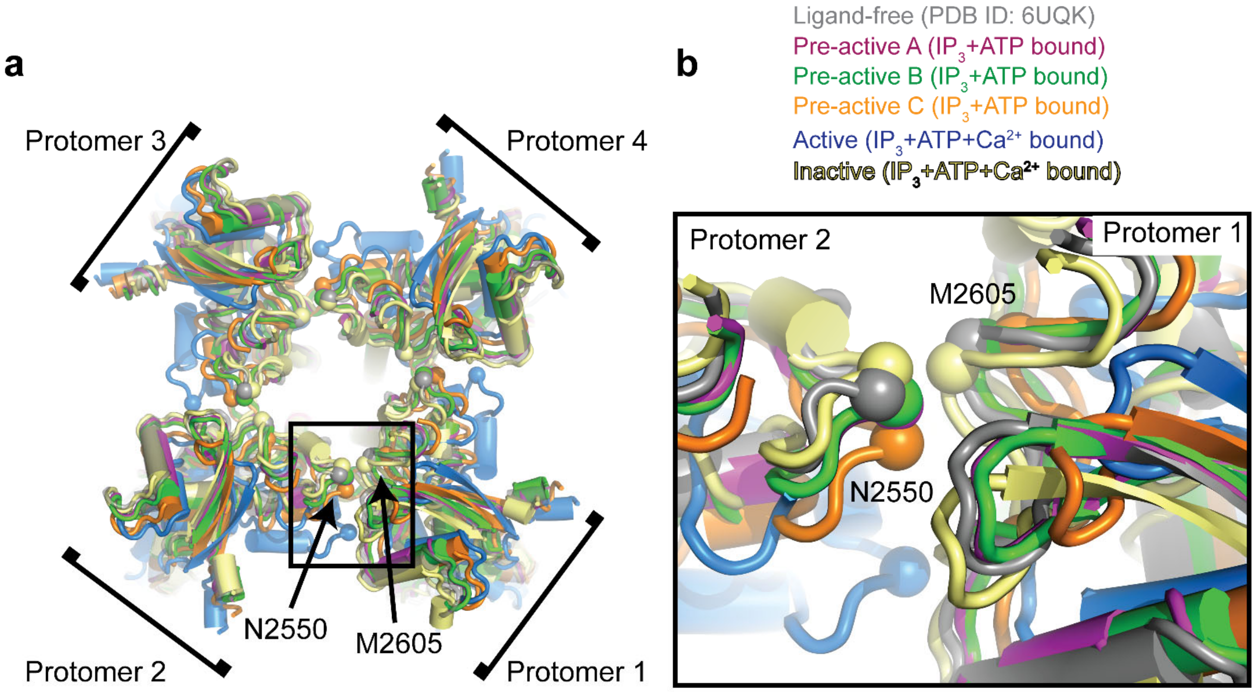

Supplementary Fig. 14: Structural changes of the JDs. a The tetrameric JDs of hIP$_3$R-3 in multiple gating conformations viewed from cytoplasm through the 4-fold symmetry axis. Structures are superposed on the residues forming the selectivity filter and P-helix of the TMDs. **b** Zoomed view of the boxed area. Structures are colored as in Fig. 2. The Cα atoms for the residues N2550 and M2605 are shown as spheres to emphasize the structural differences among different conformations.

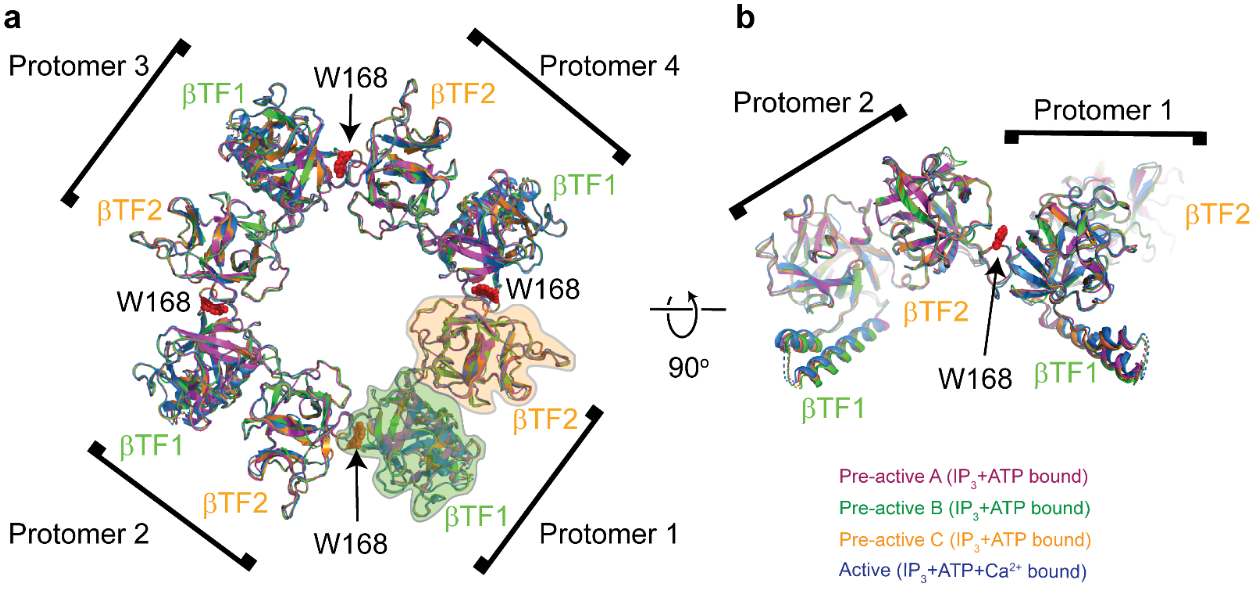

Supplementary Fig. 15: Structure of the βTF ring. a Aligned structures of the βTF rings of hIP_3R-3 in multiple gating conformations viewed from cytoplasm through the 4-fold symmetry axis. **b** 90° rotated view of panel a. Structures are colored as in Fig. 2. The βTF1 and βTF2 of each protomer are labeled, and they are circled for one of the protomers to highlight their boundaries. W168 is shown as red spheres.

Supplementary Table 1: Cryo-EM data collection, refinement, and validation statistics

**Data collection and processing**					
Microscope	FEI Krios G3i microscope				
Detector	Gatan K3 direct electron camera				
Nominal magnification	105,000 x				
Voltage (kV)	300				
Electron exposure (e/Å^2)	~60				
Defocus range (μm)	-0.8 to -1.6				
Pixel size (Å)	0.828				
Number of Micrographs	42,361				
Particles images (no.)	846,122				
Conformational state	**Pre-active A**	**Pre-active B**	**Pre-active C**	**Active**	**Inactive**
PDB ID	7T3P	7T3Q	7T3R	7T3T	7T3U
EMDB ID	EMD-25667	EMD-25668	EMD-25669	EMD-25670	EMD-25671
Symmetry imposed	C4	C4	C4	C4	C1
Final particles images (no.)	116,925	153,765	75,994	20,039	198,441
Map resolution (Å) (FSC threshold=0.143)	3.2	3.3	3.4	3.8	3.7
**Refinement**					
Model resolution (Å) (original map, FSC threshold=0.5)	3.7	3.6	3.8	4.1	8.0
Model resolution (Å) (composite map, FSC threshold=0.5)	3.0	3.0	3.1	3.6	3.6
B-factor used for map sharpening (Å^2)	-100	-100	-100	-100	-100
Model composition					
Non-hydrogen atoms	66,992	68,124	68,780	68,516	44,501
Protein residues	8,188	8,336	8,444	8,408	7,269
Zn^{2+}	4	4	4	4	4
IP_3	4	4	4	4	2
ATP	4	4	4	4	4
Ca^{2+}	-	-	-	4	4
Mean B factors (Å^2)					
Protein	46.89	63.80	67.80	113.62	79.55
Ligands	61.05	74.57	83.85	172.47	89.42
R.m.s. deviations					
Bond lengths (Å)	0.004	0.004	0.004	0.003	0.002
Bond angles (°)	0.514	0.519	0.519	0.510	0.434
Molprobity score	1.73	1.73	1.77	1.74	1.63
Clash score	8.24	7.57	8.25	8.16	6.21
Poor rotamers (%)	0.00	0.00	0.00	0.05	0.00
Ramachandran plot					
Favored (%)	95.88	95.48	95.30	95.68	95.77
Allowed (%)	4.12	4.52	4.65	4.32	4.23
Disallowed (%)	0.00	0.00	0.05	0.00	0.00

Supplementary References

1 Paknejad, N. & Hite, R. K. Structural basis for the regulation of inositol trisphosphate receptors by Ca(2+) and IP3. *Nat Struct Mol Biol* **25**, 660-668 (2018).

2 Azumaya, C. M., Linton, E. A., Risener, C. J., Nakagawa, T. & Karakas, E. Cryo-EM structure of human type-3 inositol triphosphate receptor reveals the presence of a self-binding peptide that acts as an antagonist. *J Biol Chem* **295**, 1743-1753 (2020).

3 des Georges, A. *et al.* Structural Basis for Gating and Activation of RyR1. *Cell* **167**, 145-157 e117 (2016).

4 Melville, Z., Kim, K., Clarke, O. B. & Marks, A. R. High-resolution structure of the membrane-embedded skeletal muscle ryanodine receptor. *Structure* (2021).
