# Supplementary material for: Structural basis for activation and gating of IP3 receptors
Source: Nat Commun. 2022 Mar 17;13:1408. doi: 10.1038/s41467-022-29073-2 (PMC8930994; doi:10.1038/s41467-022-29073-2)
Supplement: Supplementary file 3 — Description of Additional Supplementary Files [file 41467_2022_29073_MOESM3_ESM.pdf]

Description of Additional Supplementary Files

File Name: Supplementary Movie 1

Description: **Conformational changes during hIP$_3$R-3 activation and gating.** This video shows a morph of the hIP3R-3 structure from 'pre-active A' to 'pre-active B', 'pre-active B' to 'pre-active C', and 'preactive C' to 'active' states viewed from the side (left) and top (right). Two opposing subunits are colored as individual domains, similar to Fig. 1. The other two subunits are colored in grey. IP3 and ATP are shown as spheres and colored red. The gate-forming residues F2513 and I2517 are shown as sticks. Brackets indicate the depth of the view for the movies on the right. A green sphere is used to indicate the Ca^{2+} binding site.
