# Supplementary material for: Structural basis for activation and gating of IP3 receptors
Source: Nat Commun. 2022 Mar 17;13:1408. doi: 10.1038/s41467-022-29073-2 (PMC8930994; doi:10.1038/s41467-022-29073-2)
Supplement: Supplementary file 5 — Reporting Summary [file 41467_2022_29073_MOESM5_ESM.pdf]

nature portfolio

Corresponding author(s): Erkan Karakas

Last updated by author(s): Feb 20, 2022

Reporting Summary

Nature Portfolio wishes to improve the reproducibility of the work that we publish. This form provides structure for consistency and transparency in reporting. For further information on Nature Portfolio policies, see our Editorial Policies and the Editorial Policy Checklist.

Statistics

For all statistical analyses, confirm that the following items are present in the figure legend, table legend, main text, or Methods section.

n/a	Confirmed	
☒	☐	The exact sample size (*n*) for each experimental group/condition, given as a discrete number and unit of measurement
☒	☐	A statement on whether measurements were taken from distinct samples or whether the same sample was measured repeatedly
☒	☐	The statistical test(s) used AND whether they are one- or two-sided<br>*Only common tests should be described solely by name; describe more complex techniques in the Methods section.*
☒	☐	A description of all covariates tested
☒	☐	A description of any assumptions or corrections, such as tests of normality and adjustment for multiple comparisons
☒	☐	A full description of the statistical parameters including central tendency (e.g. means) or other basic estimates (e.g. regression coefficient) AND variation (e.g. standard deviation) or associated estimates of uncertainty (e.g. confidence intervals)
☒	☐	For null hypothesis testing, the test statistic (e.g. *F*, *t*, *r*) with confidence intervals, effect sizes, degrees of freedom and *P* value noted<br>*Give P values as exact values whenever suitable.*
☒	☐	For Bayesian analysis, information on the choice of priors and Markov chain Monte Carlo settings
☒	☐	For hierarchical and complex designs, identification of the appropriate level for tests and full reporting of outcomes
☒	☐	Estimates of effect sizes (e.g. Cohen's *d*, Pearson's *r*), indicating how they were calculated

Our web collection on statistics for biologists contains articles on many of the points above.

Software and code

Policy information about availability of computer code

Data collection	SerialEM (3.8)<br>EPU (2.10) (Thermofisher)
Data analysis	Relion 3.0.8<br>MotionCor2 (1.3.1)<br>Gtcf (1.0.6)<br>CryoSPARC v3.2.0<br>Phenix (1.20-4459)<br>Chimera (1.15)<br>ChimeraX (1.3)<br>Coot (0.9.5)<br>Pymol (v2)<br>HOLE version (2.2.005)

For manuscripts utilizing custom algorithms or software that are central to the research but not yet described in published literature, software must be made available to editors and reviewers. We strongly encourage code deposition in a community repository (e.g. GitHub). See the Nature Portfolio guidelines for submitting code & software for further information.

Data

Policy information about availability of data

All manuscripts must include a data availability statement. This statement should provide the following information, where applicable:

- Accession codes, unique identifiers, or web links for publicly available datasets
- A description of any restrictions on data availability
- For clinical datasets or third party data, please ensure that the statement adheres to our policy

Cryo-EM maps and atomic coordinates are deposited to the Electron Microscopy Data Bank (EMDB) and Protein Data Bank (PDB) databases, respectively. The accession codes are EMD-25667 [https://www.ebi.ac.uk/pdbe/entry/emdb/EMD-25667] and 7T3P [http://dx.doi.org/10.2210/pdb7t3b/pdb] for pre-active A, EMD-25668 [https://www.ebi.ac.uk/pdbe/entry/emdb/EMD-25668] and 7T3Q [http://dx.doi.org/10.2210/pdb7t3q/pdb] for pre-active B, EMD-25669 [https://www.ebi.ac.uk/pdbe/entry/emdb/EMD-25669] and 7T3R [http://dx.doi.org/10.2210/pdb7t3r/pdb] for pre-active C, EMD-25670 [https://www.ebi.ac.uk/pdbe/entry/emdb/EMD-25670] and 7T3T [http://dx.doi.org/10.2210/pdb7t3t/pdb] for active, and EMD-25671 [https://www.ebi.ac.uk/pdbe/entry/emdb/EMD-25671] and 7T3U [http://dx.doi.org/10.2210/pdb7t3u/pdb] for inactive states, respectively. Reagents and other materials will be available upon request from E.K. with a completed materials transfer agreement.

The following previously published cryo-EM maps and atomic coordinates were used;

EMDB ID: EMD-20849 [https://www.ebi.ac.uk/pdbe/entry/emdb/EMD-20849], Cryo-EM structure of type 3 IP3 receptor revealing presence of a self-binding peptide.

PDB ID:6UQK [http://dx.doi.org/10.2210/pdb6uqk/pdb], Cryo-EM structure of type 3 IP3 receptor revealing presence of a self-binding peptide.

PDB ID:6DRC [http://dx.doi.org/10.2210/pdb6drc/pdb], High IP3 Ca2+ human type 3 1,4,5-inositol trisphosphate receptor.

PDB ID:6DQV [http://dx.doi.org/10.2210/pdb6dqv/pdb] Class 2 IP3-bound human type 3 1,4,5-inositol trisphosphate receptor.

PDB ID: 5TAL [http://dx.doi.org/10.2210/pdb5tal/pdb], Structure of rabbit RyR1 (Caffeine/ATP/Ca2+ dataset, class 1&2).

PDB ID:7M6A [http://dx.doi.org/10.2210/pdb7m6a/pdb], High resolution structure of the membrane embedded skeletal muscle ryanodine receptor.

Field-specific reporting

Please select the one below that is the best fit for your research. If you are not sure, read the appropriate sections before making your selection.

☒ Life sciences ☐ Behavioural & social sciences ☐ Ecological, evolutionary & environmental sciences

For a reference copy of the document with all sections, see nature.com/documents/nr-reporting-summary-flat.pdf

Life sciences study design

All studies must disclose on these points even when the disclosure is negative.

Sample size	846,122 single particle images from 42,261 micrographs were used for 3D reconstructions. No statistical methods were used to determine sample size for EM data.
Data exclusions	Particles were excluded based on the 2D and 3D classifications.
Replication	Datasets collected on 4 different grids prepared from the same purification prep were highly similar and combined for the final analysis.
Randomization	The datasets were randomly split in two halves which were refined independently. Resolution was assessed based on the Fourier shell correlation between the half maps at the 0.143 threshold criteria.
Blinding	Blinding is not applicable to this study since the samples investigated are required to be known.

Reporting for specific materials, systems and methods

We require information from authors about some types of materials, experimental systems and methods used in many studies. Here, indicate whether each material, system or method listed is relevant to your study. If you are not sure if a list item applies to your research, read the appropriate section before selecting a response.

Materials & experimental systems

n/a	Involved in the study
☒	☐ Antibodies
☐	☒ Eukaryotic cell lines
☒	☐ Palaeontology and archaeology
☒	☐ Animals and other organisms
☒	☐ Human research participants
☒	☐ Clinical data
☒	☐ Dual use research of concern

Methods

n/a	Involved in the study
☒	☐ ChIP-seq
☒	☐ Flow cytometry
☒	☐ MRI-based neuroimaging

Eukaryotic cell lines

Policy information about cell lines

Cell line source(s)	Sf9 insect cells from ThermoFisher (Catolog number: 12659017)
Authentication	none of the cell lines were authenticated.
Mycoplasma contamination	No test was performed
Commonly misidentified lines (See ICLAC register)	No commonly misidentified cell lines were used in the study.
